# Draft Genome Sequence of the Psychrophilic Gliding Species *Gelidibacter algens* ACAM 536

**DOI:** 10.1128/genomeA.00908-16

**Published:** 2016-09-01

**Authors:** John P. Bowman

**Affiliations:** Tasmanian Institute of Agriculture, University of Tasmania, Sandy Bay, Tasmania, Australia

## Abstract

A draft genome sequence was obtained from the type strain of *Gelidibacter algens* (ACAM 536). This species was isolated from sea-ice diatom assemblages collected from Ellis Fjord, Eastern Antarctica. The genome of ACAM 536 is a single circular chromosome with an estimated size of 4.50 Mbp.

## GENOME ANNOUNCEMENT

*Gelidibacter algens* was first isolated from coastal sea-ice core slices possessing distinct algal assemblages (1). The ice cores were collected from Ellis Fjord, Prydz Bay, Eastern Antarctica (68°36′S 78°5′E). The type strain is ACAM 536 (= C8ST5 = ATCC 700364). *G. algens* belongs to the family *Flavobacteriaceae* (order Flavobacteriales, class *Flavobacteriia*, phylum *Bacteroidetes*) and is a strictly aerobic, yellow pigmented, rod-shaped psychrophilic halophile that moves by gliding and is able to utilize a range of carbohydrates and organic acids. Best growth occurs on marine agar between 15 and 18°C. The yellow pigment formed is the carotenoid zeaxanthin.

To generate a draft genome sequence, high-molecular-weight DNA was obtained using a modified Marmur method (1). DNA was sequenced using the 454 GS-FLX/Plus (454 Life Sciences, Branford, CT, USA) platform following the manufacturer’s *de novo* sequencing protocol. A total of 137 Mb of sequence data (430- to 440-bp average length) was assembled using Newbler version 2.8 (454 Life Sciences), yielding 140 contigs (*N*_50_, 88 kb; *L*_50_, 13 contigs). Genome coverage was estimated to be 30-fold.

The genome data was annotated using Prodigal (2), RAST (3), and the NCBI Prokaryotic Genome Annotation Pipeline (http://www.ncbi.nlm.nih.gov/genome/annotation_prok). The resulting annotation was manually inspected for consistency as well as to functionally assign open reading frames. RAST and tRNA-scan (4) were used to delineate tRNA and rRNA coding regions.

The genome of *G. algens* ACAM 536 consists of a single circular chromosome with an estimated size of 4.50 Mbp and a G+C content of 37.2 mol%. From the genome data, 3,840 coding sequences (CDSs), 3,657 genes, and 181 pseudogenes could be defined. The genome has 2 rRNA operons, 38 tRNA genes, and 4 ncRNAs. These dimensions are consistent with the draft genome of *G. mesophilus* (DOE Joint Genome Institute, AUHD00000000.1), which has a size of 4.42 Mb, a G+C content of 36.8 mol%, 3,795 CDSs, 3 rRNA operons, and 36 tRNA genes.

Interesting features on the *G. algens* ACAM 536 genome include genes for proteorhodopsin and its cognate β-carotene monooxygenase. Two genetic regions populated by nonribosomal peptide synthetases (NRPSs) and polyketide synthases (PKSs) are noted. The first contained two NRPS genes, while the second contains a type I PKS gene polyketide synthase and NPRS genes also found in *G. mesophilus*. A large cluster of genes include pathways for polysaccharide metabolism. The genome also contains many genes associated with oxidative stress management, including 5 Dps-like iron-binding proteins, consistent with the species dwelling in an environment supersaturated with oxygen.

### Accession number(s).

This whole-genome shotgun project has been deposited in DDBJ/ENA/GenBank under the accession number LZRN00000000. The version described in this paper is the first version, LZRN01000000.
